# 220. A Phase 2 Double-Blind, Placebo-Controlled Study Showing Oral Tableted Norovirus Vaccine VXA-G1.1-NN is Immunogenic, Efficacious, and Reduces Viral Shedding Following Norovirus Challenge

**DOI:** 10.1093/ofid/ofae631.078

**Published:** 2025-01-29

**Authors:** Susan Greco, Becca A Flitter, Lam Nguyen, Maria D Apkarian, Elena D Neuhaus, Darreann Carmela Hailey, Sean N Tucker, James F Cummings

**Affiliations:** Vaxart, Inc, South San Francisco, California; Vaxart Inc, South San Francisco, California; Vaxart Inc, South San Francisco, California; Vaxart Inc., Phoenix, Arizona; Vaxart, San Francisco, California; Vaxart, Inc., South San Francisco, California; Vaxart, San Francisco, California; VAXART, South San Francisco, California

## Abstract

**Background:**

Norovirus (NV) is a leading cause of acute gastroenteritis worldwide. Currently no specific therapy exists for norovirus gastroenteritis (NVG). VXA-G1.1-NN is a nonreplicating adenovirus-vectored thermostable oral NV vaccine shown in clinical trials to be safe, well-tolerated and generates robust serum and mucosal immune responses. This study investigated safety, immunogenicity, and protective efficacy of VXA-G1.1-NN following NV GI.1 challenge.

**Figure 1.**
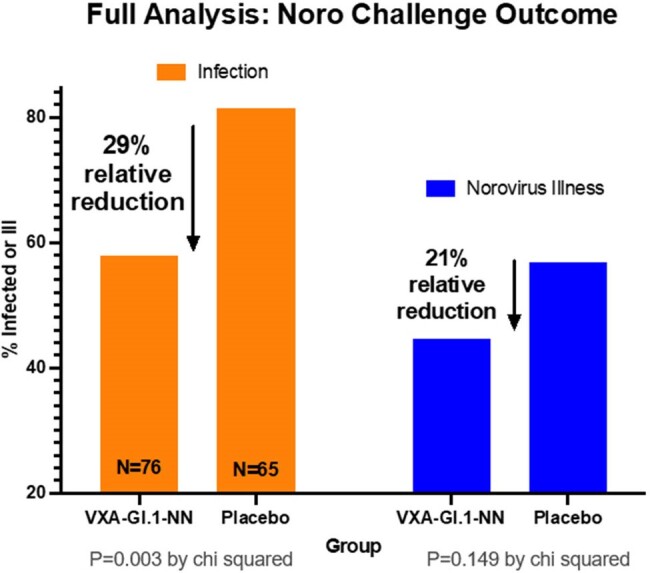
Clinical outcomes (norovirus infection and norovirus gastroenteritis) after norovirus challenge 28 days post vaccination with VXA-G1.1-NN or placebo

**Methods:**

165 healthy adults ages 18-49 were randomized 1:1 to a single oral vaccination of VXA-GI.1-NN or placebo. At 28 days post-vaccination, 141 eligible subjects were challenged with 1x10^6^ genomic copies NV GI.1 inoculum. Solicited symptoms of reactogenicity were recorded for 1 week after vaccination and unsolicited adverse events (AEs) through 28 days post challenge. NVG was assessed by incidence of acute gastroenteritis (AGE) with evidence of NV+ infection. NV shedding was evaluated by qPCR in stool and emesis. VP1-specific GI.1 IgA antibody secreting cells (ASC), serum IgA and IgG, and Norovirus Blocking Antibody Assay (NBAA) were assessed. Totality of evidence analysis was used to assess overall vaccine protective effect.

**Figure 2.**
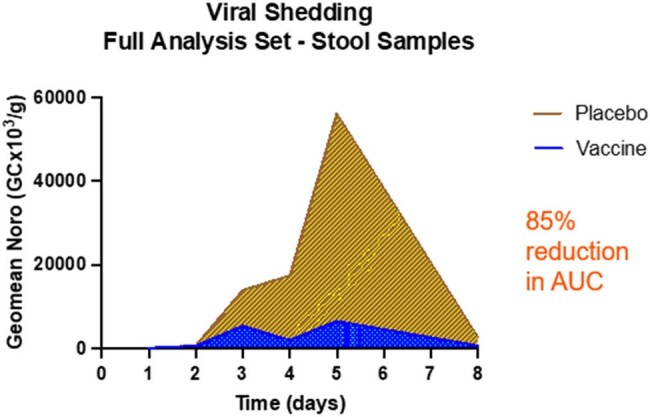
Viral shedding in stool by qPCR through 7 days post challenge with GI.1 NN virus inoculum following vaccination with VXA-G1.1-NN or placebo

**Results:**

VXA-G1.1-NN was safe and well tolerated.VXA-G1.1-NN induced strong serum and cellular immunogenicity with substantial increases in VP1 GI.1- specific ASC and serum antibodies. Serum functional antibody responses, determined by NBAA, were significantly higher in subjects in the vaccine group compared to placebo. VP1 specific IgA was significantly increased in nasal secretions and saliva in the vaccine group. Protective efficacy for prevention of NVG was 21% (p= 0.149) and for NV infection was 29% (p= 0.003). An 85% decrease in geometric mean viral shedding in stool in the VXA-G1.1-NN group was also observed. Totality of evidence analysis for all 6 outcomes simultaneously provided a z-score of 5.56 (p< 0.001), indicating protective benefit of VXA-G1.1-NN.

**Figure 3.**
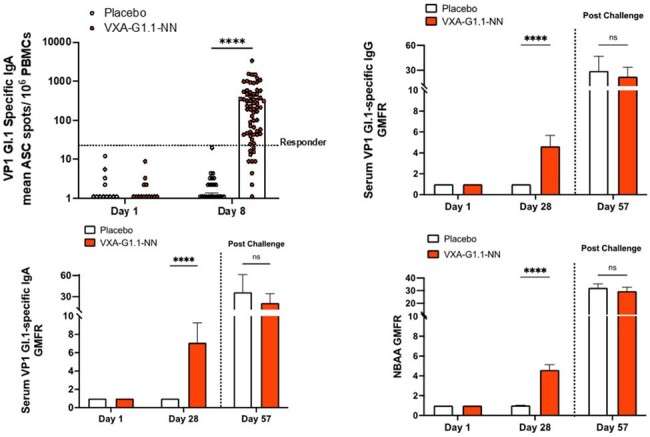
Cellular and serum immune responses following vaccination with VXA-G1.1-NN or placebo

**Conclusion:**

VXA-G1.1-NN was safe, immunogenic, and reduced both viral shedding and NV infection following NV challenge. VXA-G1.1-NN tableted vaccine may help limit outbreaks by reducing viral shedding. Totality of evidence analysis for treatment effect provided a strong statistical signal supporting a protective effect of VXA-G1.1-NN.

**Disclosures:**

**Susan Greco, MD, MPH**, Vaxart, Inc: Stocks/Bonds (Public Company) **Becca A. Flitter, PhD, MPH**, Vaxart Inc: Stocks/Bonds (Public Company) **Lam Nguyen, MD**, CVS Health: Stocks/Bonds (Public Company)|Vaxart: Author is either employed and/or has received stock options from Vaxart as part of this work.|Vaxart: Stocks/Bonds (Public Company) **Darreann Carmela Hailey, MS**, Bionano Genomics: Stocks/Bonds (Public Company)|Vaxart, Inc.: Stocks/Bonds (Public Company) **Sean N. Tucker, PhD**, Vaxart, Inc.: Grant/Research Support|Vaxart, Inc.: patent|Vaxart, Inc.: Ownership Interest|Vaxart, Inc.: Stocks/Bonds (Public Company) **James F. Cummings, MD**, VAXART: Stocks/Bonds (Public Company)

